# Antioxidant Activity and Structural Characterization of Anthocyanin–Polysaccharide Complexes from *Aronia melanocarpa*

**DOI:** 10.3390/ijms252413347

**Published:** 2024-12-12

**Authors:** Jie Wang, Jingyi Wang, Jiahui Hao, Miao Jiang, Congcong Zhao, Ziluan Fan

**Affiliations:** 1 College of Life Science, Northeast Forestry University, 26 Hexing Road, Xiangfang District, Harbin 150040, China; wj18365936610@163.com (J.W.); owo_guyu@163.com (J.W.); haojiahui5832@163.com (J.H.); xyxm_jm@163.com (M.J.); 15689447928@163.com (C.Z.); 2 Key Laboratory of Forest Food Resources Utilization, Harbin 150040, China

**Keywords:** *Aronia melanocarpa*, anthocyanins, polysaccharides, antioxidant activity, interaction

## Abstract

Anthocyanins and polysaccharides are among the primary components of numerous foodstuffs, and their interaction exerts a considerable influence on the texture and nutritional value of foods. In order to improve the antioxidant properties and stability of anthocyanins as well as their bioavailability, in this study, anthocyanin–polysaccharide complexes with varying compounding ratios (1:0.5, 1:1.0, 1:1.5, 1:2.0, 1:2.5, 1:3.0) were prepared from *Aronia melanocarpa* anthocyanins and polysaccharides derived from the fruit pomace of *Aronia melanocarpa*. These compounds were characterized, and their antioxidant capacity was determined. The findings demonstrated that the antioxidant activity of anthocyanins was markedly enhanced through the process of compounding with polysaccharides. The most efficacious antioxidant effect was determined by measuring the IC_50_ of the antioxidant activity of mixtures at different anthocyanin/polysaccharide complexing ratios. The results of ultraviolet–visible spectroscopy, infrared spectroscopy, and scanning electron microscopy revealed the features of the anthocyanin–polysaccharide complexes with ratios of 1:0.5, 1:1.0, 1:1.5, and 1:2.5. The anthocyanins and polysaccharides were observed to enhance the intensity of ultraviolet absorption with respect to that of the individual molecules, and it was noted that they were able to bond to each other through hydrogen bonding. Additionally, the morphology of the compositions differed from that of the individual components. This provides a theoretical foundation for the structural design of anthocyanin–polysaccharide-containing foods and the development and utilization of novel food ingredients.

## 1. Introduction

Anthocyanins are a class of flavonoids [1] and are water-soluble natural colorants that occur naturally in a range of fruits and vegetables, including pitaya, blueberries, and purple cabbage. Additionally, some minor berries, including *Vaccinium vitis-idaea* [2], *Aronia melanocarpa* [3], and *Lonicera edulis* [4], contain anthocyanin components with considerable antioxidant capacity. The benefits of anthocyanins to the human body are manifold. In addition to their antioxidant properties, they inhibit enzyme activity [5], such as that of α-glucosidase and α-amylase, slowing down glycolysis and preventing diabetes, thereby reducing the risk of cardiovascular disease [6]. Furthermore, they possess anti-radiation [7] and anti-inflammatory effects [8], aligning with the contemporary societal demand for natural, safe, and healthy products [9].

*Aronia melanocarpa* (Michx.) EII, also known as wild cherry berry or ageless berry, belongs to the Rosaceae family, genus Aronia, and is a perennial deciduous shrub. Its origin is in the north-eastern part of North America, and it is distributed from the coast of the Baltic Sea to the Pacific Ocean. It was introduced into the semi-arid area of north-western Liaoning Province for planting [10]. *Aronia melanocarpa* has distinctive functional attributes and exceptional health benefits, aligning with the concept of ‘medicine and food’ homology. Its abundant polyphenols facilitate the removal of free radicals and heavy metals from the body, inhibit oxidation, and bolster the functionality of the human immune system [11]. Approximately 26% of the polyphenolic compounds found in *Aronia melanocarpa* are anthocyanins [12], which possess strong antioxidant properties [13]. Additionally, they have anti-ageing [14], anti-cancer [15], and anti-inflammatory [16] properties and have been linked to the prevention of urinary tract infections [17]. Furthermore, they have been demonstrated to be effective in the treatment of diabetes [18] and obesity [19]. Their efficacy in the prevention and treatment of cardiovascular and cerebrovascular diseases has also been established [6]. However, as with other anthocyanins, *Aronia melanocarpa* anthocyanins are unstable and susceptible to factors such as temperature and environmental conditions [20]. Furthermore, they are seldom consumed due to the sour taste of the fruits [21], which reduces their bioavailability [22].

A substantial corpus of literature exists that demonstrates that anthocyanins can bind to other substances, thereby enhancing their stability and bioavailability. For example, Koh [23] found that chelator-soluble pectin extracted from blueberries prevented the degradation of anthocyanins more effectively than water-soluble pectin and exhibited higher in vitro stability. Mulberry polysaccharides, which comprise a high proportion of galacturonic acid, were extracted at elevated temperatures and pressures, resulting in a notable enhancement in the bioaccessibility of cornflavin-3-O-glucoside within the small intestine [24]. The binding of pectin and anthocyanins can be enhanced under high-pressure conditions due to the impact of high pressure on non-covalent interactions, such as hydrogen bonding and hydrophobic interactions. This maintains the antioxidant properties of anthocyanins and improves their stability [25]. The thermal [26] and color [27] stability of anthocyanins is enhanced by grape pectin polysaccharides, primarily through hydrophobic interactions. Three types of polysaccharides demonstrated the capacity to retain the color, stability, and antioxidant capacity of anthocyanins under heat treatment, with XG exhibiting the greatest retention (XG > KGM > BG) [28]. It has been demonstrated that when polyphenols are physically mixed with polysaccharides, non-covalent interactions, including hydrogen bonding, hydrophobic interactions, and ionic interactions, result in the formation of reversible or irreversible complexes between the polyphenols and the polysaccharides. These covalent interactions are mediated by the enzymatic or non-enzymatic oxidation of o-quinone [29].

**Figure 1:**
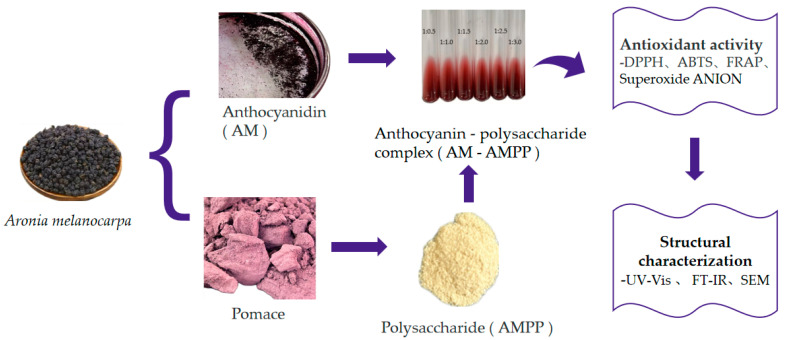
The experimental scheme of this study.

*Aronia melanocarpa* has been found to have a higher polysaccharide content than both wild blueberries and indigo fruits, reaching up to 1.764 mg/g [30]. Polysaccharides have the capacity to scavenge free radicals within the organism [31] and demonstrate favorable biological activities, including anti-inflammatory [32] an immunomodulatory [33] activities, regulatory effects on the intestinal flora [34], and anti-radiation [35] properties. The pomace of *Aronia melanocarpa* also contains polysaccharide components [36], which were extracted following the anthocyanin extraction. The experimental scheme of this study is shown in Figure 1.

In this study, the anthocyanins and polysaccharides of *Aronia melanocarpa* were selected for analysis. The interactions between anthocyanins and polysaccharides were analyzed using a combination of UV–visible spectroscopy, infrared spectroscopy, and scanning electron microscopy. The impact of varying anthocyanin-to-polysaccharide ratios on the microstructure and surface characteristics of anthocyanin–polysaccharide complexes, as well as the antioxidant efficacy of these complexes, were examined. In comparison to the utilization of other anthocyanin and polysaccharide compounds, the use of polysaccharide components present into fruit pomace for compounding not only enhances the value of the pomace, but also facilitates its efficient reuse. This practice is not only aligned with the contemporary concept of green and sustainable development, but also mitigates the environmental impact of waste. This research contributes to our understanding of the physicochemical properties of anthocyanin–polysaccharide complexes in food systems and provides new ideas and possibilities for the development of functional foods and nutraceuticals.

## 2. Results

### 2.1. Polysaccharide Characterization

**Figure 2:**
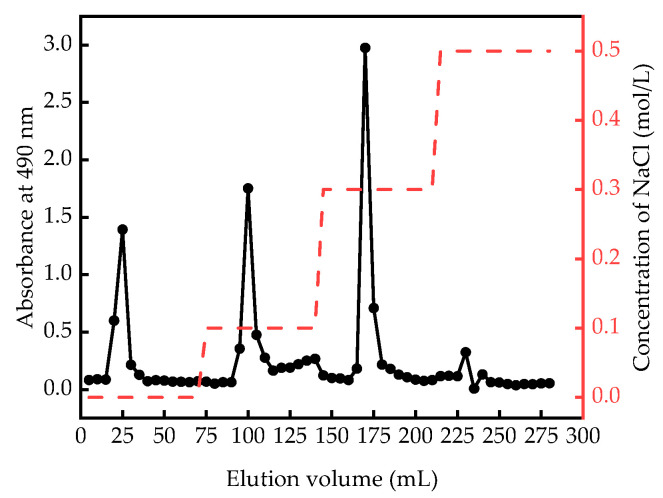
Elution curve of AMPPs.

The elution curve of the polysaccharides from *Aronia melanocarpa* is shown in Figure 2.

The DEAE-52 cellulose column is an anion-exchange column that separated the samples into distinct fractions following the application of progressively diluted sodium chloride solutions (0, 0.1, 0.3, 0.5 mol/L) in a stepwise manner. The elution curve from DEAE-52 cellulose column chromatography of the crude polysaccharides of *Aronia melanocarpa* is illustrated in Figure 2, which depicts four distinct peaks. The high-content eluate components were subsequently collected, resulting in the isolation of a white powdered polysaccharide sample through concentration, dialysis, and freeze-drying.

**Figure 3:**
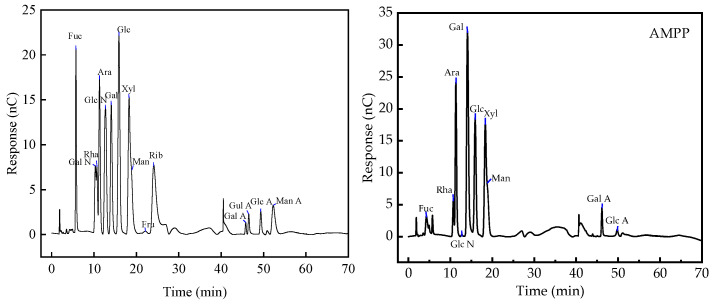
Ionic chromatograms of 16 mixed standard sugars and the polysaccharides from *Aronia melanocarpa*.

The analysis in Figure 3 demonstrated that the polysaccharides of *Aronia melanocarpa* primarily included fucose, rhamnose, arabinose, galactose, glucose, xylose, and mannose, at a molar ratio of 0.012:0.072:0.107:0.206:0.071:0.123:0.074, with the contents of galactose and galacturonide being higher. This indicated that the polysaccharides of *Aronia melanocarpa* were probably acidic in nature. This result is in accordance with that of Dong [37], although the ratios differed slightly.

The molecular weights of the two fractions obtained for the polysaccharides of *Aronia melanocarpa* may be attributed to the utilization of disparate concentrations of NaCl to elute the mixture of polysaccharides. The weight-average molecular weight (Mw) of the two polysaccharide fractions was found to be 51,089 and 21,214 Da, the number-average molecular weight (Mn) was 50,584 and 20,644 Da, and the polydispersity index (PDI) values (Mw/Mn) were found to be 1.010 and 1.028, respectively.

### 2.2. Antioxidant Activity

The mass concentration of anthocyanins was maintained at 4 mg/mL at pH 3.0, and the concentration of polysaccharides was varied to determine the scavenging ability of each sample against the DPPH radical and ABTS radical, its ferric ion reduction, and the scavenging ability against superoxide anion radicals. The scavenging ability was determined using Vc as a positive control.

**Figure 4:**
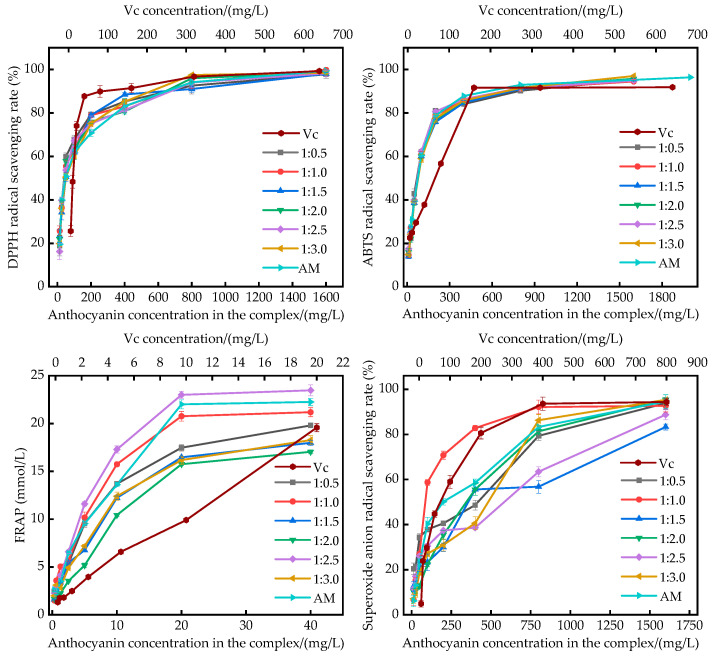
Antioxidant activities against DPPH, ABTS, and superoxide anion and FRAP of anthocyanin and polysaccharide complexes from *Aronia melanocarpa*.

As illustrated in Figure 4, the complexes exhibited notable scavenging capabilities against DPPH radicals, ABTS radicals, and superoxide anion radicals and robust iron reduction potential. Free radicals are generated as a consequence of normal metabolic processes. However, the excessive accumulation of free radicals can cause oxidative damage to the organism, which may contribute to the development of age-related diseases such as cancer and other chronic conditions [38]. DPPH is a relatively stable free radical and has been widely used as a marker to assess the free radical scavenging ability of various antioxidants. Figure 4 illustrates the DPPH free radical scavenging abilities of anthocyanins, polysaccharides, and their complexes. Figure 4 illustrates that as the concentration of anthocyanin and polysaccharide complexes increased, their DPPH scavenging ability also rose. However, all of the complexes exhibited a lower scavenging effect than Vc. At concentrations below 400 mg/L, it was evident that the complexes exhibited a higher scavenging rate against DPPH free radicals than anthocyanins. At the concentration of 200 mg/L, the scavenging rate of anthocyanins against the DPPH radicals was relatively low. However, the antioxidant activity of the complexes increased significantly, from 70.82% to 75.11–79.32%, in comparison to the activity of anthocyanins alone when polysaccharides were added at varying concentrations. At a concentration of 400 mg/L, the rate of DPPH scavenging for all the complexes and anthocyanins reached values of over 80%, and then gradually stabilized. Furthermore, the scavenging ability of anthocyanins for DPPH radicals was also enhanced gradually. The order of the scavenging ability of the compositions against the DPPH radicals, as indicated by the IC_50_ value, was as follows: 1:1.0 > 1:0.5 > 1:2.0 > 1:1.5 > 1:2.5 > 1:3.0.

ABTS, an organic radical reagent, is frequently employed to evaluate the antioxidant activity of natural products. ABTS radicals generated from an ABTS solution in the presence of potassium persulfate exhibit a pronounced absorption peak in the visible region [39]. As illustrated in Figure 4 the ABTS radical scavenging capacity of the complexes demonstrated an increase with the elevation of anthocyanin concentration. However, the scavenging capacity of all complexes remained inferior to that of Vc. The complexes exhibited varying scavenging capacities depending on their ratio. The order of the complexes’ ABTS radical scavenging capacities was as follows: 1:2.5 > 1:1.0 > 1:0.5 > 1:3.0 > 1:2.0 > 1:1.5. The 1:2.5 complex exhibited the strongest ABTS radical scavenging capacity, with an IC_50_ value of 63.46 mg/L, in contrast to the other complexes, which demonstrated a weaker capacity. The 1:1.5 complex exhibited the highest ABTS radical scavenging capacity, with an IC_50_ value of 63.46 mg/L. Conversely, the 1:1.5 complex exhibited the lowest ABTS radical scavenging capacity, with an IC_50_ value of 70 mg/L. Above a concentration of 80 mg/L, the ABTS radical scavenging rate of anthocyanins exhibited a slight improvement in comparison to that of each complex. At concentrations between 10 and 80 mg/L, the scavenging ability of anthocyanins against ABTS was in the middle range compared to that of the mixtures. At concentrations lower than 5 mg/L, the anthocyanin ABTS radical scavenging capacity was observed to be superior to that of the individual complexes, a result that is consistent with the findings of Rui [40].

The FRAP method is founded upon the premise that an antioxidant reduces the complex (Fe^3^⁺-TPTZ) to the ferrous form (Fe^2^⁺-TPTZ), thereby generating a blue color with maximum absorption at 593 nm [41]. The FRAP capacities of anthocyanins and complexes were evaluated, and the results are presented in Figure 4 As illustrated in the figure, the FRAP values exhibited an increase with the elevation in complex concentration. The complex at a 1:2.5 ratio demonstrated the most robust antioxidant capacity for ferric ions, followed by the 1:1.0 complex. The antioxidant capacity of the complexes against ferric ions was in the range of 1.49 to 23.47 mmol/L. Upon reaching a concentration of 20 mg/L, the FRAP values of the complexes exhibited a gradual stabilization, while the FRAP values of the anthocyanins demonstrated a slight decline. The FRAP values of the anthocyanins (22.0064–23.4722 mmol/L) were only lower than those of the complexes at a 1:2.5 compounding ratio (22.9967–23.4722 mmol/L), with a difference of approximately 1 mmol/L between the two types of antioxidants.

Superoxide radicals represent the most prevalent free radical species that engage in direct or indirect attacks on a range of biomolecules, including carbohydrates, lipids, proteins, and nucleic acids [42]. Figure 4 illustrates the scavenging capacity against superoxide anion radicals of complexes with varying compounding ratios. In the concentration range of 0–1600 mg/L, the complexes exhibited enhanced scavenging capacity against superoxide anion radicals, with the scavenging rate increasing from 6.602% to 97.79% as the concentration rose. Nevertheless, at equivalent mass concentrations, the scavenging of superoxide anion radicals by Vc was more effective than the scavenging effect of the complexes and anthocyanins. At concentrations exceeding 100 mg/L, the scavenging ability of the complex with a compound ratio of 1:1.0 exhibited a markedly higher efficacy than that of the other complexes. At a concentration of 1600 mg/L, the scavenging of superoxide anion by anthocyanins (94.579%) was higher than that of the other complexes, with the exception of the complex with a 1:2.0 ratio. This indicated that the scavenging ability of anthocyanins themselves was not inferior to that of the complexes at high concentration. Furthermore, at a low concentration, the combination of anthocyanins with polysaccharides could markedly enhance the intrinsic antioxidant capacity of anthocyanins.

The DPPH radical scavenging and superoxide anion radical scavenging were most pronounced for the complex with a ratio of 1:1.0, as illustrated in Table 1. The ABTS radical scavenging was most prominent for the complex with a ratio of 1:2.5, followed by the 1:1.0 complex. The ferric reducing antioxidant power (FRAP) values of the complexes are illustrated in Figure 4, which demonstrates that the complex with a ratio of 1:2.5 exhibited the greatest capacity to scavenge ferric ions, followed by the complex with a ratio of 1:1.0. The superoxide anion radical scavenging ability of the complex with a ratio of 1:1.0 was significantly higher than that of the complex with a ratio of 1:2.5. Therefore, it can be concluded that the complex with a ratio of 1:1.0 had the strongest antioxidant ability.

### 2.3. Thermal Stability Analysis of the Complexes

The impact of varying temperatures on anthocyanin retention within the complexes was investigated at a solution pH of 3, with pure anthocyanins serving as a control.

**Figure 5:**
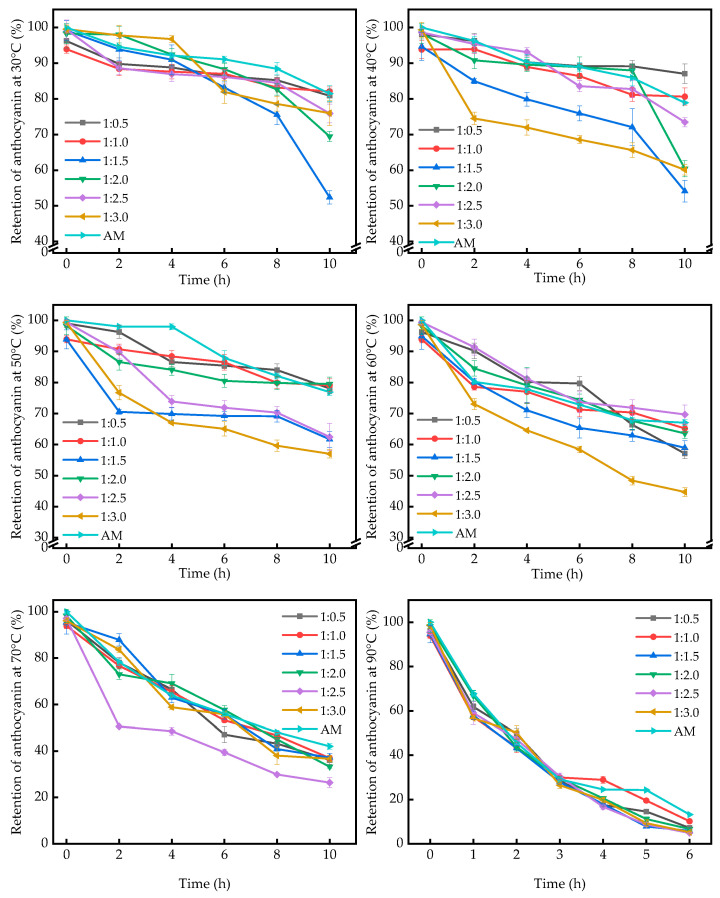
Anthocyanin retention in complexes at different temperatures.

Figure 5 illustrates that the overall variation trend of the complexes and anthocyanins was identical, with both undergoing degradation throughout the experiment. The temperature range of 30–50 °C revealed no significant distinction between the anthocyanins and their respective complexes. At 40 °C, the anthocyanins in pure form exhibited a marginally higher retention compared to the complexes, which persisted until the 6th hour of treatment. At 30 °C, the retention of anthocyanins in the complexes with anthocyanin-to-polysaccharide addition ratios of 1:0.5, 1:1.0, 1:1.5, 1:2.0, 1:2.5, and 1:3.0 at 6 h was 86.38 ± 1.66%. The retention of anthocyanins was found to be 87.00 ± 1.30%, 83.16 ± 1.54%, 88.31 ± 1.66%, 86.14 ± 1.98%, and 81.93 ± 3.22%, while the retention of pure anthocyanins (91.08 ± 0.87%) was higher than that of the complexes. This phenomenon may be attributed to the reaction between anthocyanins and polysaccharides, which results in the encapsulation of anthocyanins by polysaccharides, thereby reducing their exposure [43]. The trend observed at temperatures ranging from 40 to 50 °C was comparable to that observed at 30 °C.

The retention rate of anthocyanins in the complexes was observed to be higher than that in pure anthocyanins at 60 °C. The retention rate of anthocyanins at 50 °C decreased from 98.01 ± 0.54% to 80.18 ± 0.62% after 2 h. At this point in time, anthocyanins have begun to undergo degradation. For the complexes at ratios of anthocyanins to polysaccharides of 1:0.5, 1:1.5, 1:2.0, 1:2.5, 1:2.0, 1:2.5 and 1:2.5, the retention rate of anthocyanins was higher than that for pure anthocyanins. The retention rate of anthocyanins in the 1:2.5 composition was higher than that of pure anthocyanins, which can be attributed to the degradation of the polysaccharides at this temperature. This resulted in the release of the encapsulated anthocyanins, leading to a higher content of anthocyanins in the solution of the composition.

At a temperature of 70 °C, the retention in the complexes decreased to approximately 40% after 10 h, whereas at 90 °C, this process was completed in only 2 h. After 6 h, the retention was below 20% in all cases. The 90 °C water bath environment was observed to degrade the anthocyanins at a more rapid rate than that observed at 70 °C. The anthocyanin retention of the anthocyanin solution without added polysaccharide was 24.54 ± 0.50% after 4 h at 90 °C. For the samples with ratios of anthocyanins to polysaccharides of 1:0.5, 1:1.0, 1:1.5, 1:2.0, 1:2.5, after 4 h at 90 °C, the retention rates were 17.53 ± 0.53%, 28.88 ± 1.63%, 17.93 ± 0.33%, 20.52 ± 0.68%, 16.72 ± 1.15%, and 19.82 ± 1.12%, respectively. The interaction between anthocyanins and polysaccharides has been demonstrated to enhance the thermal stability of anthocyanins to a certain extent. However, the degradation of anthocyanin–polysaccharide complexes still occurs at high temperatures. This is attributed to the inherent instability of polysaccharides at elevated temperatures, which reduces their protective effect [44] and results in a convergence of the retention rates of anthocyanins in anthocyanin solutions and compositions after prolonged treatment at temperatures of 60, 70, and 90 °C. The retention rates of anthocyanins in anthocyanin solutions and mixtures are a consequence of prolonged exposure to elevated temperatures. The protective effect of polysaccharides on anthocyanins is also diminished under prolonged high-temperature conditions. This indicates that polysaccharides can delay the rapid degradation of anthocyanins during high-temperature treatment, but only within a certain time frame. Therefore, it is advisable to avoid prolonged high temperatures during the extraction and processing of anthocyanins [45].

### 2.4. UV-Vis Spectral Analysis

**Figure 6:**
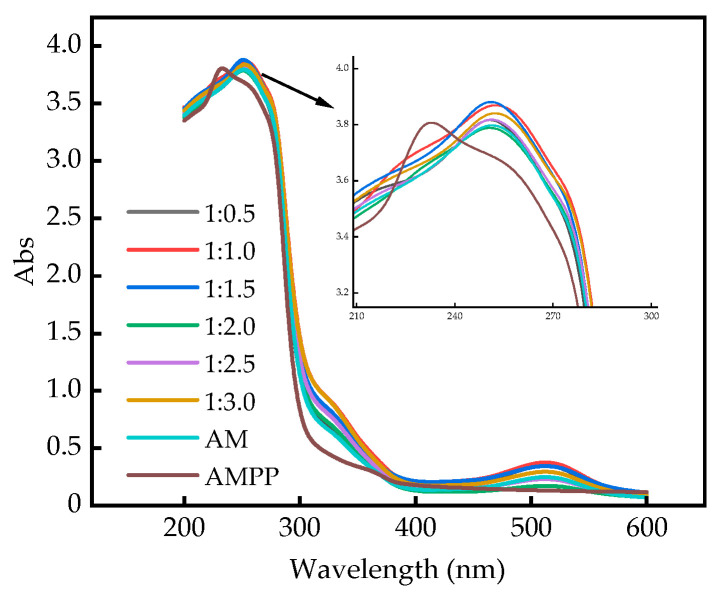
UV–visible absorption spectra.

In order to investigate the effect of different ratios of polysaccharides on anthocyanins, the UV-Vis absorption spectra of the complexes, anthocyanins, and polysaccharides were measured in citrate-phosphate buffer at pH 3.0. The UV-Vis spectra of anthocyanins, polysaccharides, and complexes are presented in Figure 6.

The results demonstrated that anthocyanins exhibited an absorption peak at 250 nm, a low absorption peak at 510 nm in the visible region, and no absorption peak at 300–330 nm. This indicates that these anthocyanins lacked acyl groups [46]. The UV–visible spectrum of the complexes exhibited similarities to that of anthocyanins, displaying absorption peaks at 250 nm and 510 nm. Furthermore, some complex ratios demonstrated higher absorption intensities than anthocyanins and polysaccharides. The pure polysaccharides exhibited a single absorption peak at 230 nm and displayed enhanced smoothness at 250 nm and 510 nm. However, the complexes with different ratios of polysaccharides mixed with anthocyanins exhibited varying degrees of redshift, indicating potential structural alterations in the molecules within the complexes. Similar observations of a redshift were reported by Fernandes [47], following the interaction of wine anthocyanins with commercial citrus pectin. No notable change was observed in the complex at 510 nm. The extent of the color-enhancing effect of the complexes was indicated by the increase in absorbance observed in the range of 200–600 nm. At 250 nm, the absorption values of all the complex groups were higher than that of anthocyanins, with the exception of the complex with a ratio of 1:2.0, which exhibited significantly lower absorption values than anthocyanins. This may be attributed to the absorption of UV light by the molecules in the complexes, the energy level jump of their electrons, and the increase in the electron conjugation system, which resulted in the occurrence of a color-enhancing effect. Alternatively, it may be due to the fact that the anthocyanin molecules may be adsorbed on the polysaccharides. This is consistent with the findings of Wang [24], Dong [28], Fernandes [48], and others.

### 2.5. FT-IR Spectral Analysis

**Figure 7:**
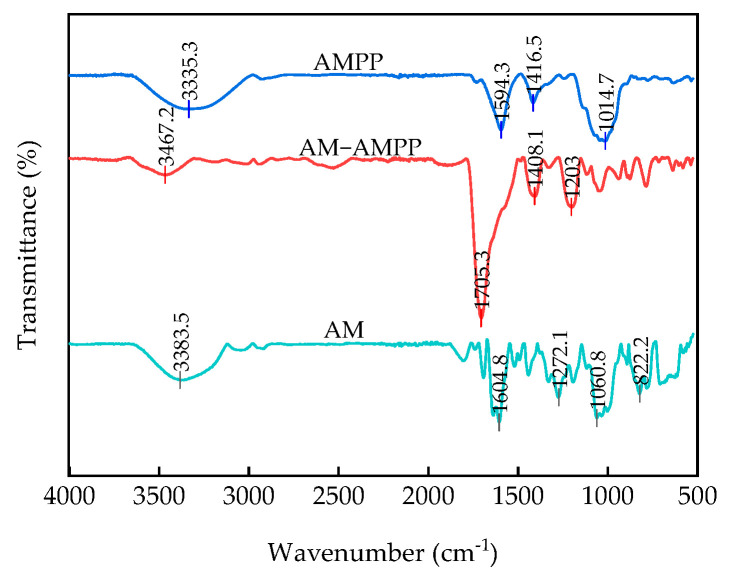
Infrared spectrogram. AM, anthocyanin; AMPP, polysaccharide; AM-AMPP, anthocyanin–polysaccharide complex.

The FTIR spectrograms of all the samples were recorded in the range of 500 to 4000 cm^−1^ under the wave number shown in Figure 7.

For polysaccharides, a broad absorption peak at 3335.3 cm^−1^ was observed, which was caused by intra- or intermolecular O-H stretching vibrations characteristic of galacturonic acid in the polysaccharide molecule [49]. A weaker absorption peak was observed near 2930.1 cm^−1^, which was attributed to the C-H stretching vibration on the sugar chain. This peak is regarded as one of the characteristic peaks of polysaccharides [50]. The peak observed at 1594.3 cm^−1^ can be attributed to the presence of glyoxalate in the polysaccharides, which exhibits a C=O stretching vibration characteristic of glyoxalate. The absorption peak at 1416.5 cm^−1^ was attributed to the variable-angle vibration of the CH bond, and is also regarded as a characteristic peak of polysaccharides [50]. The absorption peak observed in the range of 1200–1000 cm^−1^ was attributed to the stretching vibration of the C-O-H side group and the C-O-C glycosidic bond. No notable absorption peaks were evident at 1540 cm^−1^ in the IR spectrogram, indicating that the polysaccharides no longer contained a protein component. This finding is consistent with the observations of Jin [51].

The broader absorption peak at 3383.5 cm^−1^ indicated the presence of a certain amount of hydrogen bonding in the anthocyanins, while the peak at 1680–1450 cm^−1^ was attributed to the C=C bond. The presence of a double bond stretching vibration is also indicated by a broader peak at 1604.8 cm^−1^, along with two smaller peaks near 1500 cm^−1^. This evidence supports the conclusion that anthocyanins do indeed contain a benzene ring structure [52]. The peaks at 1272.1 cm^−1^ and 1060.8 cm^−1^ were attributed to the bending of the aromatic ring C-H group and the stretching of the pyran ring in flavonoids, thereby substantiating the presence of a pyran ring structure in anthocyanins [28]. The absorption peak at 822.2 cm^−1^ may be attributed to unsaturated-C-H-bond out-of-plane deformation vibrations.

The spectra of all anthocyanin–polysaccharide complexes exhibited a high degree of similarity, while the positions of the OH absorption peaks in the anthocyanin and polysaccharide spectra exhibited a notable deviation from those observed for the complexes. The lower peaks at 3350–3330 cm^−1^ and near 1500 cm^−1^ in the complexes’ spectra in comparison to those of anthocyanins and polysaccharides might be attributed to the interaction of the hydroxyl groups in the polysaccharides with the hydroxyl groups in the anthocyanins. The formation of hydrogen bonds between anthocyanins and polysaccharides results in a reduction in the number of free hydroxyl groups [53,54]. Furthermore, the benzene rings and hydroxyl groups present in the anthocyanins facilitate non-covalent interactions, which are responsible for the formation of the complexes. The gradual enhancement of the spectral bands of the anthocyanin–polysaccharide complexes at 1710–1411 cm^−1^, particularly at 1705.3 cm^−1^, provided further evidence for the formation of interactions between anthocyanins and polysaccharides.

### 2.6. SEM Analysis

In scanning electron microscopy (SEM), the surface of a sample is scanned by an electron beam, allowing the surface topography to be obtained with a resolution of less than 1 nm [55].

**Figure 8:**
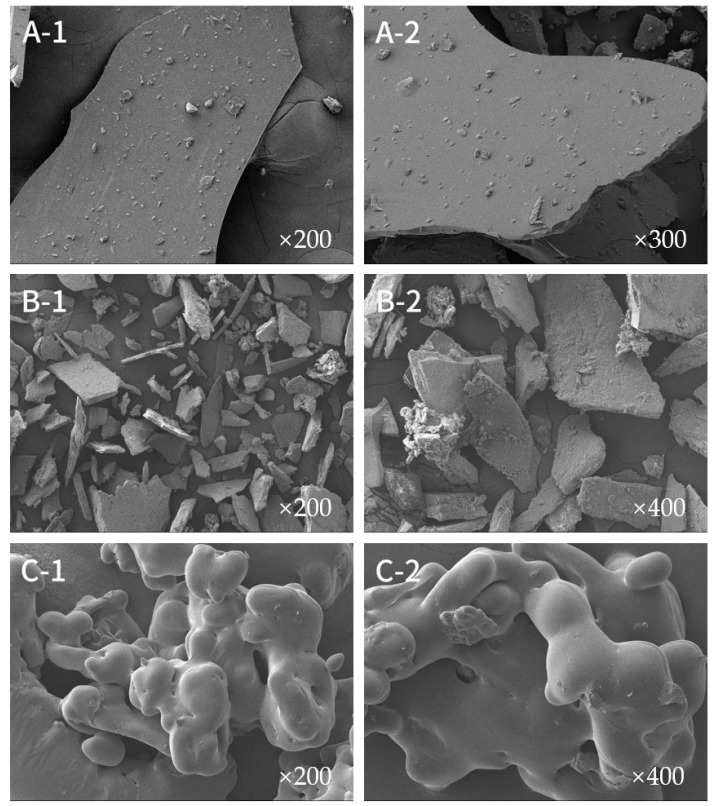
Scanning electron microscopy images at different magnifications. ((**A-1**,**A-2**) Anthocyanins; (**B-1**,**B-2**) polysaccharides; (**C-1**,**C-2**) complexes).

Figure 8 illustrates the scanning electron microscopy (SEM) images of the anthocyanins and polysaccharides of *Aronia melanocarpa* and their complexes. As illustrated in Figure 8(A-1,A-2), the anthocyanins exhibited a smooth, scale-like morphology with some elevated surfaces, likely resulting from the attachment of small molecules to larger molecules. The anthocyanins were dispersed in varying sizes and fragments, a finding that aligns with the results reported in a previous study [25].

As illustrated in Figure 8(B-1,B-2), the polysaccharides exhibited a lamellar structure with varying dimensions and dispersed configurations, accompanied by the presence of irregular particles. At 400× magnification, the rough surface was observed to be more rugged, with the presence of debris. The observation of a porous and flocculent structure in some of the granular objects indicated the potential for repulsive forces between the polysaccharide molecules, suggesting that the intermolecular forces of attraction might be relatively weak [56].

Figure 8(C-1,C-2) illustrates that the scanning electron micrographs of anthocyanin–polysaccharide complexes exhibited a more pronounced morphological alteration, resulting in the formation of larger amorphous particles with no discernible angles on the surface. This phenomenon may be attributed to the formation of a strong intermolecular attraction between the polysaccharides and the anthocyanins, which led to the stacking of particles and the development of larger amorphous structures. The formation of these larger particles can be attributed to the strong attraction between polysaccharides and anthocyanin molecules [28], as well as to the solubility of both polysaccharides and anthocyanins in water. An aqueous medium facilitates the adsorption of anthocyanins onto macromolecular biopolymers [43], resulting in the observed particle growth. Furthermore, the complexes displayed smooth, loose, highly porous, and highly adherent reticular surfaces, which may be consistent with the high viscosity results observed for these particles. The smooth surfaces of these complexes may indicate that the anthocyanins were uniformly solubilized and solidified in the polysaccharide biopolymers. This may be due to the fact that these biopolymers consisted of amorphous polysaccharides containing vacancies [43]. This study provides further evidence that anthocyanins can tightly bind to polysaccharides.

The anthocyanin and polysaccharide complexes demonstrated superior antioxidant properties compared to the individual components, suggesting a potential for their use in scavenging free radicals. The above conclusion is further corroborated by Figure 5, which illustrates that the scavenging ability of different free radicals varied with different compounding ratios. This paper presents the findings that anthocyanin–polysaccharide complexes with a compound ratio of 1:1.0 exhibited the optimal antioxidant properties. Furthermore, *Aronia melanocarpa* is rarely consumed due to its sour taste, which results in the loss of numerous beneficial active ingredients. The polysaccharides in this study were extracted and purified from the pomace of *Aronia melanocarpa* following the extraction of anthocyanins, thereby enhancing the utilization of *Aronia melanocarpa*.

## 3. Discussion

The binding mechanism between anthocyanins and polysaccharides is contingent upon a number of factors, including their structural characteristics, molecular weight, and physicochemical properties. The interaction between anthocyanins and polysaccharides is achieved in two steps. Initially, the hydroxyl groups on the surface of the polysaccharides form a rigid structure with water, while hydrophobic cavities or voids are formed inside the polysaccharides. Subsequently, anthocyanins are driven into the cavities or voids through hydrophobic interactions [57]. The above binding mechanism was further confirmed by scanning electron microscopy as well as infrared experiments, which provided an experimental basis for the binding of anthocyanins with polysaccharides.

The combination of anthocyanins from *Aronia melanocarpa* and polysaccharides from the fruit pomace offers significant advantages in terms of resource utilization, functional enhancement, and biostability. The complexes were demonstrated to enhance the ability of anthocyanins to scavenge free radicals, improving their scavenging efficiency and reducing oxidative stress. They have a promising application in food, nutraceuticals, and cosmetics, particularly in the fields of anti-ageing and anti-inflammation research and cardiovascular protection. However, there are a number of challenges in relation to technology, cost, and product quality consistency. Although polysaccharides can enhance the stability of anthocyanins, anthocyanins may still be degraded under prolonged storage or extreme processing. Furthermore, it is difficult to avoid their color change and reduction in biological activity. The low content of polysaccharides in fruit pomace makes the extraction and purification process complex and costly, which increases the overall production cost and affects market competitiveness. Additionally, food safety requirements must be met.

Furthermore, these results provide a scientific basis for the application of the described complexes in food and cosmetics. Qin [58] incorporated an anthocyanin-rich purple corn extract (PCE) and silver nanoparticles (AgNPs) into a chitosan film, which demonstrated robust antibacterial efficacy against Escherichia coli, Salmonella, Staphylococcus aureus, and Listeria monocytogenes. Bai [59] demonstrated that the combination of Nigella sativa polysaccharides and grape seed proanthocyanidins could mitigate the detrimental effects of radiation on splenocyte DNA. This was achieved by reducing the levels of oxidation products and enhancing the antioxidant capacity, thereby conferring a significant radioprotective effect. Further investigation into the antibacterial and anti-UV properties of anthocyanin–polysaccharide complexes may be conducted at a later stage. In light of the preliminary evidence suggesting the potential antibacterial and antiradical properties of the anthocyanin–polysaccharide complexes, these areas warrant further investigation. Bacteriostatic experiments can facilitate a more nuanced understanding of the complexes’ inhibitory effect on diverse microbial species, as well as their prospective applications in food preservation, medical dressings, and other domains. Radiation resistance experiments can elucidate their capacity to shield cells from radiation-induced damage, which is of paramount importance for the advancement of novel protective materials and the enhancement of safety protocols for astronauts and radiation workers.

It is therefore imperative that in the future, a greater number of interdisciplinary researchers be brought together to conduct a comprehensive assessment of anthocyanin–polysaccharide complexes in terms of their stability, bacteriostatic potential, and radiation resistance. This assessment should be conducted using advanced experimental techniques and methods, with a view to establishing a solid scientific foundation for their wide application in a variety of fields, such as food, medicine, and aerospace.

## 4. Materials and Methods

### 4.1. Materials

*Aronia melanocarpa* was purchased from Changbai Mountain (Changchun, China). DPPH and TPTZ were purchased from Biotopped (Beijing, China). ABTS was purchased from Yuanye (Shanghai, China). All other chemicals and reagents used were of analytical grade.

### 4.2. Extraction and Purification of Anthocyanidins from Aronia melanocarpa

Extract the anthocyanins from *Aronia melanocarpa* in an ultrasonic water bath. Accurately weigh 100.00 g of frozen fruit of *Aronia melanocarpa* using an electronic balance, add 95% ethanol in a ratio of 1:20, and then add 10 mL of acetic acid. Homogenize (BME 100 L, Weiyu, Shanghai, China) in a tissue grinder for 5 min, remove the homogenate from the grinder, and use ultrasound-assisted extraction. The extraction parameters were the following: extraction solvent, 95% ethanol; ultrasonic power, 200 W; extraction temperature, 45 °C; and ultrasonic time, 37 min [60]. The mixture after ultrasound treatment was centrifuged at 4500 rpm for 10 min and then filtered. The supernatant was concentrated after centrifugation using a vacuum rotary evaporator at 40 °C and kept for further purification [61]. The filter residue was dried for later use.

The crude extract of anthocyanins was purified using the X-5 macroporous resin. The specific steps of purification were as follows: soak the macroporous resin in ethanol for 24 h, rinse with distilled water until there is no alcohol odor, then soak in a 10% acetic acid solution for 12 h, rinse with deionized water until neutral, add a 5% NaOH solution to soak the resin for 12 h, rinse with deionized water until neutral, and then load a column. The crude extract of anthocyanins was loaded onto the column at a concentration of 100 mg/mL. After complete adsorption, it was washed with distilled water until the outlet was free of viscosity. The eluent was replaced to wash and collect the anthocyanins (using 60% ethanol as the eluent). Subsequently, the solution was concentrated by vacuum rotary evaporation at 40 °C until the alcoholic flavor was removed and finally dried to obtain the purified anthocyanin product, namely, AM (*Aronia melanocarpa* anthocyanin). The content of anthocyanins in *Aronia melanocarpa* was determined to be 16.665 g/100 g using the pH differential method [62].

### 4.3. Extraction and Determination of Aronia melanocarpa Polysaccharides

Extract the polysaccharides from the fruit pomace using the hot water extraction method. The extraction parameters were as follows: material to liquid ratio of 1:15, extraction temperature of 90 °C, and extraction time of 4 h. The extracted supernatant was obtained by centrifugation, concentrated, and precipitated with an 80% ethanol solution to obtain crude polysaccharides from *Aronia melanocarpa* [30].

The crude polysaccharides were purified using a DEAE-52 cellulose column. The purification sequence was as follows: first, the crude polysaccharides were decolorized using the hydrogen peroxide decolorization method and then, they were deproteinized using Savage reagent (chloroform-to-n-butanol volume ratio of 4:1). After evaporation and concentration, the decolorized, deproteinized polysaccharides were obtained. After dissolving the polysaccharides, they were dialyzed using a dialysis bag for 48 h to remove excess reagents. After evaporation, concentration, and drying, the polysaccharides to be loaded onto the column were obtained.

After pretreatment, DEAE-52 cellulose was directly soaked in an appropriate amount of deionized water to remove any suspended impurities. Then, the column was loaded by the wet method and washed with twice the volume of deionized water to compact it. Dissolve the polysaccharides from *Aronia melanocarpa* in water and prepare a 10 mg/mL aqueous solution for adsorption on the column, with a sample volume of 20 mL. Then, use three times the volume of the column of distilled water and 0.1, 0.3, and 0.5 mol/L NaCl solutions for fractional elution, with a flow rate set at 1 mL/min. Collect 5 mL from each elution, and detect the polysaccharide efflux content using the phenol sulfuric acid method. Collect and combine the concentrated solutions of polysaccharides corresponding to single peak positions. Put the mixture into a dialysis bag, dialyze for 48 h, freeze-dry, and obtain the pure polysaccharides of *Aronia melanocarpa*, namely, AMPP (pure polysaccharide from *Aronia melanocarpa*).

The polysaccharides of *Aronia melanocarpa* were subjected to a detailed analysis, with the polysaccharide content determined by the sulfuric acid–phenol method [63], resulting in a value of 244.04 mg/g. Following the method of Wang [64], the content of uronic acid was determined to be 26.75% using a UV–visible spectrophotometer (UV-5500PC, METASH, Shanghai, China). The molecular weight (Mw) was determined by high-performance gel permeation chromatography. The monosaccharide composition of the polysaccharides was determined by ion chromatography [65].

### 4.4. Preparation of Anthocyanin–Polysaccharide Complexes

The combination of anthocyanins and purified polysaccharides from *Aronia melanocarpa* was carried out according to the method described in reference [66]. Dissolve the anthocyanin powder (4 mg/mL) in a buffer solution and mix it with the polysaccharides in different proportions to achieve varying polysaccharide concentrations. The mass ratios of anthocyanins to polysaccharides were 1:0.5, 1:1.0, 1:1.5, 1:2.0, 1:2.5, and 1:3.0. The dispersion of anthocyanins and polysaccharides should be achieved through magnetic stirring, after which the mixture should be stored at 4 °C to prevent light exposure. The resulting compound, AM-AMPP (anthocyanin–polysaccharide complex from *Aronia melanocarpa*), can then be freeze-dried for subsequent use.

### 4.5. Antioxidant Properties of the Complexes

#### 4.5.1. Determination of the Clearance of the DPPH Radical

The preparation of the DPPH working solution was carried out according to the method described in reference [67]. The DPPH working solution was prepared by accurately weighing 6.00 mg of DPPH powder and diluting it with a 95% ethanol solution to a volume of 100 mL, while thoroughly mixing. Then, 100 μL of the gradient mass concentration sample solutions was added to the wells of a 96-well plate along with 200 μL of the 100 μmol/L DPPH ethanol solution, thoroughly mixed, and reacted in the dark for 30 min, with the absorbance A_i_ measured at a wavelength of 517 nm. For the control group, a 95% ethanol solution was used in place of the DPPH solution, and the absorbance A_j_ was measured. For the blank group, a 95% ethanol solution was used in place of the sample, and the absorbance A_0_ was measured. The DPPH free radical scavenging rate of the samples was calculated according to the following formula

$$\mathrm{DPPH}\;\mathrm{radical}\;\mathrm{scavenging}\;\mathrm{activity}\left(\%\right)=\left(1-\frac{A_{i}-A_{j}}{A_{0}}\right)\times 100$$

#### 4.5.2. Determination of the Clearance of the ABTS Radical

The preparation of the ABTS working solution was carried out according to the method described in reference [68]. To prepare the ABTS radical working solution, accurately weigh 0.384 g of ABTS radical powder and dissolve it, obtaining 10 mL of ABTS solution, accurately weigh 0.134 g of potassium persulfate and dissolve it, obtaining 100 mL of potassium persulfate solution, mix the two reagents in a 1:1 ratio, and keep the mixture in the dark for 12 h. Dilute it 40 times with distilled water before use. Sequentially add the following in a 96-well plate: for Group A_i_, gradient concentration sample solutions, 40 µL, and ABTS radical working solution, 160 µL, mix well; for Group A_j_, gradient concentration sample solutions, 40 µL, and distilled water, 160 µL, mix well; and for Group A_0_ (blank group), distilled water, 40 µL, and ABTS radical working solution, 160 µL, mix well. Let all groups react in the dark for 6 min, use ascorbic acid as a positive control, measure the absorbance at 734 nm wavelength, and conduct three repeated experiments for each sample, each with three replicates. Calculate the scavenging ability of the ethanolic extracts against the ABTS radical free radical according to the following formula

$$\mathrm{ABTS}\;\mathrm{radical}\;\mathrm{scavenging}\;\mathrm{activity}\left(\%\right)=(1-\frac{A_{i}-A_{j}}{A_{0}})\times 100$$

#### 4.5.3. Determination of the Iron Reduction Antioxidant Capacity (FRAP Method)

The method proposed by WEN [69] was used to determine the antioxidant capacity based on iron reduction, with slight modifications. Prepare a TPTZ working solution (25 mL, pH 3.6, of acetate buffer, 2.5 mL of a 10 mM TPTZ solution, 2.5 mL of 20 mM ferric chloride). Mix 100 μL of the sample solutions with 4 mL of the TPTZ working solution. After heating at 37 °C for 15 min, measure the mixture absorbance at a wavelength of 593 nm.

Drawing of the standard curve: Take 0.1 mL of a series of ferrous sulfate solutions at the concentrations of 0, 0.1, 0.2, 0.4, 0.6, 0.8, and 1.0 mmol/L, add 0.3 mL of deionized water, mix well, and then add 3.0 mL of the FRAP working solution. After mixing well, react in a 37 °C water bath for 4 min, measure the mixture absorbance at 593 nm, and plot the results, with absorbance on the vertical axis and ferrous sulfate solution concentration on the horizontal axis. The standard curve equation is y = 0.1517x + 0.1081 (R^2^ = 0.9994).

#### 4.5.4. Superoxide Anion Radical Scavenging Ability

The method of WU [70] was used to determine the scavenging rate of superoxide anion radicals. Take 4.5 mL of a Tris HCl buffer solution with a pH of 8.2 and add it to a test tube. Leave it at room temperature for 25 min. Then, add 1 mL of sample diluent and Vc solution with different mass concentrations and 0.3 mL of 3 mmol/L pyrogallol (prepared with 10 mmol/L LHCL), mix well, and react at 25 °C for 5 min. Finally, immediately add 1 mL of 8 mol/L HCl to stop the reaction. Measure the absorbance value at 325 nm wavelength and record it as A_1_; measure the absorbance value using 10 mmol/L HCl instead of pyrogallol and denote it as A_2_; replace the sample with distilled water to measure the absorbance value and denote it as A_0_. Using Vc as the positive control, the formula for calculating the O_2_^−^ radical scavenging rate is as follows

$$\mathrm{Superoxide}\;\mathrm{anion}\;\mathrm{radical}\;\mathrm{scavenging}\mathrm{rate}/\%=\left(1-\frac{A_{1}-A_{2}}{A_{0}}\right)\times 100$$

### 4.6. Thermal Stability of the Complexes

The prepared compound solution and the anthocyanin solution were placed at 30 °C, 40 °C, 50 °C, 60 °C, 70 °C and 90 °C, and the anthocyanin content was determined at 2 h intervals between 30 °C and 70 °C and at 1 h intervals between 70 °C and 90 °C using the pH differential method.

### 4.7. UV-Vis Spectroscopy

Scan the prepared complex solutions with a UV–visible spectrophotometer and detect the spectral changes of 6 groups of samples, as well as those of anthocyanins and pure polysaccharides, in the wavelength range of 200–600 nm at pH 3.0.

### 4.8. Fourier Infrared Spectroscopy

Use Fourier transform microscopy infrared spectrometry to analyze the structural changes of the samples. Place the freeze-dried anthocyanin–polysaccharide complex in a Fourier transform infrared micro-spectrometer (Nicolet Nexus 670 FTIR, Thermo Fisher Scientific, Waltham, MA, USA) for measurement according to the following parameter settings: 32 scans at a resolution of 32 cm^−1^, with wave number range conditions of 4000–500 cm^−1^ [29].

### 4.9. Scanning Electron Microscopy (SEM)

The SEM morphology of freeze-dried platinum samples after sputtering was observed using a scanning electron microscope (FEI Nova NanoSEM, JEOL, Tokyo, Japan). The appropriate image magnification was selected, and the acceleration voltage was 20 kV [28].

### 4.10. Statistical Analysis

Each experiment was repeated 3 times, and the results are expressed as mean ± standard deviation. We used Excel 2019 and Origin 2024 software for chart drawing and IBM SPSS Statistics 25 (IBM, New York, NY, USA) statistical software to perform one-way ANOVA on the data, with a significance level set to *p* < 0.05.

## 5. Conclusions

In this study, anthocyanins from *Aronia melanocarpa* and polysaccharides from the fruit pomace were combined through physical methods to prepare anthocyanin–polysaccharide complexes. The polysaccharides were found to contain seven monosaccharides (fucose, rhamnose, arabinose, galactose, glucose, xylose, and mannose), with molecular weights of 51,089 and 21,214 Da, respectively. The antioxidant activity of the complexes was evaluated at different ratios, and the results indicated that the complex with a 1:1.0 ratio exhibited the best antioxidant properties.

The anthocyanin–polysaccharide complexes exhibited a distinct absorption peak at 250 nm at pH 3.0, similar to that observed in the UV spectrum of anthocyanins. The complexes showed a red shift to some extent, accompanied by a color enhancement effect. In comparison to anthocyanins and polysaccharides, the infrared spectrum of the complexes revealed a decrease in peak intensity of the peak around 3330–3350 cm^−1^. The SEM images showed a significant difference between the complexes and their individual components. The complexes appeared as smooth, non-angular, amorphous particles, while the anthocyanins and polysaccharides displayed flaky or granular structures. These observations suggest that the interactions between anthocyanin and polysaccharide molecules lead to structural changes, with the formation of their complex primarily driven by hydrogen bonding or hydrophobic interactions.

## Figures and Tables

**Table 1:** IC_50_ values of complexes at different compounding ratios and anthocyanins.

IC_50_ Values/(mg/L)	1:0.5	1:1.0	1:1.5	1:2.0	1:2.5	1:3.0	Anthocyanins	Vc
DPPH	45.49 ± 5.58	45.19 ± 4.66	49.96 ± 4.87	46.02 ± 2.13	50.24 ± 4.87	52.74 ± 2.48	52.54 ± 3.84	10.27 ± 0.53
ABTS	66.24 ± 1.67	65.96 ± 2.63	69.99 ± 1.73	69.43 ± 2.47	63.48 ± 2.81	68.98 ± 2.22	67.50 ± 1.24	12.09 ± 0.75
Superoxide Anion	169.84 ± 13.05	100.22 ± 3.36	371.60 ± 18.06	255.28 ± 8.46	321.64 ± 16.51	273.16 ± 7.04	180.42 ± 4.98	55.94 ± 1.09

## Data Availability

The data presented in this study are available on request from the corresponding author.
